# Circulating tumor DNA analysis in the era of precision oncology

**DOI:** 10.18632/oncotarget.27418

**Published:** 2020-01-14

**Authors:** Rabih Said, Nicolas Guibert, Geoffrey R. Oxnard, Apostolia M. Tsimberidou

**Affiliations:** ^1^Department of Investigational Cancer Therapeutics, Phase I Clinical Trials Program, The University of Texas MD Anderson Cancer Center, Houston, TX, USA; ^2^Department of Oncology, St. George Hospital University Medical Center, University of Balamand, Beirut, Lebanon; ^3^Lowe Center for Thoracic Oncology, Dana-Farber Cancer Institute, Boston, MA, USA; ^4^Thoracic Oncology, Toulouse University Hospital, Toulouse, France; ^*^Co-authorship

**Keywords:** circulating tumor DNA analysis, clinical trials, targeted therapy, genomic profiling

## Abstract

The spatial and temporal genomic heterogeneity of various tumor types and advances in technology have stimulated the development of circulating tumor DNA (ctDNA) genotyping. ctDNA was developed as a non-invasive, cost-effective alternative to tumor biopsy when such biopsy is associated with significant risk, when tumor tissue is insufficient or inaccessible, and/or when repeated assessment of tumor molecular abnormalities is needed to optimize treatment. The role of ctDNA is now well established in the clinical decision in certain alterations and tumors, such as the epidermal growth factor receptor (EGFR) mutation in non-small cell lung cancer and the v-Ki-ras2 kirsten rat sarcoma viral oncogene homolog (KRAS) mutation in colorectal cancer. The role of ctDNA analysis in other tumor types remains to be validated. Evolving data indicate the association of ctDNA level with tumor burden, and the usefulness of ctDNA analysis in assessing minimal residual disease, in understanding mechanisms of resistance to treatment, and in dynamically guiding therapy. ctDNA analysis is increasingly used to select therapy. Carefully designed clinical trials that use ctDNA analysis will increase the rate of patients who receive targeted therapy, will elucidate our understanding of evolution of tumor biology and will accelerate drug development and implementation of precision medicine. In this article we provide a critical overview of clinical trials and evolving data of ctDNA analysis in specific tumors and across tumor types.

## INTRODUCTION

Non-invasive circulating tumor DNA (ctDNA) genotyping is a cost-effective alternative to tumor biopsies when these biopsies are associated with significant risk, tumor tissue is insufficient or inaccessible, and/or serial assessment of tumor molecular abnormalities is needed to optimize treatment. ctDNA analysis of epidermal growth factor receptor (EGFR) in non-small cell lung cancer (NSCLC) and v-Ki-ras2 kirsten rat sarcoma viral oncogene homolog (KRAS) in colorectal cancer (CRC) is well established [1, 2]. However, validation studies of the clinical relevance of ctDNA in other tumor types [3] are lacking.

**Figure 1:**
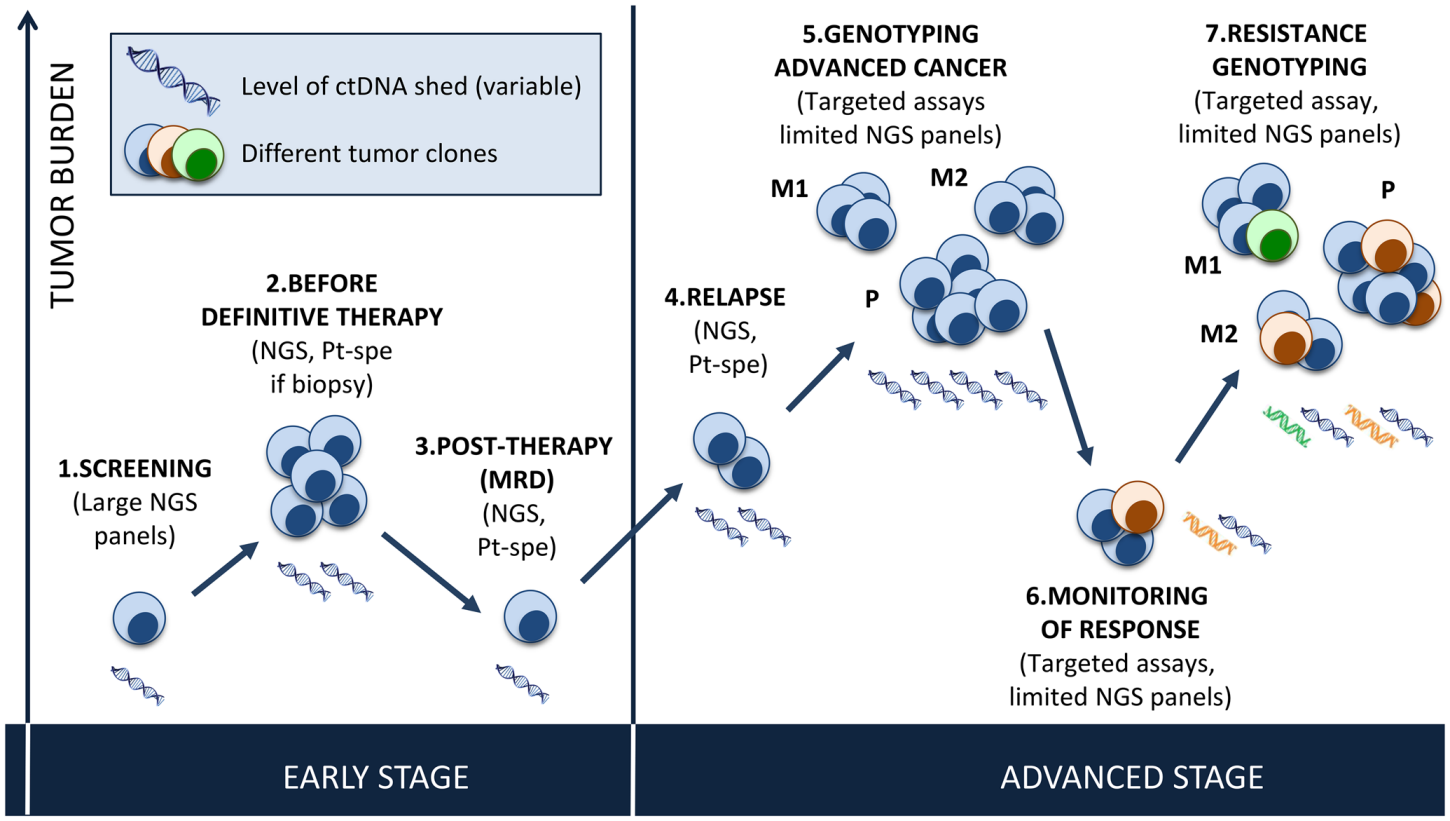
Potential use of plasma genotyping in cancer. Early stage disease: *Screening* will require the use of large *NGS* panels, with both high sensitivity and perfect specificity. *Before surgery*, determination of tumor burden in plasma has the potential to help guide neo-adjuvant or adjuvant therapy and monitor response, using large panels or patient-specific assays based on the molecular profile of the tissue biopsy when available. *After surgery*, NGS (large gene panels or patient-specific assays) can detect MRD and guide adjuvant therapy (early detection) or detect relapse. Low tumor shed in plasma will be the main limitation to the integration of plasma genotyping in early stage disease. Advanced stage disease: *At diagnosis*, ctDNA can guide genotype-directed therapy (using targeted assays focusing on a predefined gene of interest (i. e. *EGFR* in NSCLC) or targeted NGS covering genes of interest). The variations in allelic fractions allow for monitoring of treatment response, which may be helpful for pharmacodynamics analyses in phase I studies. *When acquired resistance* to targeted therapies occurs, ctDNA can detect specific mechanisms of resistance (targeted assay like for *EGFR* T790M or targeted NGS), taking into consideration the different clones present within the primary tumor (P) and all metastatic sites (M1, M2), and guide treatment adjustments.

In this systematic review, we summarize the published trials of ctDNA analysis by tumor type and across tumor types, and we discuss the role of ctDNA analysis in selecting patients for enrollment in clinical trials and in guiding targeted therapy. The potential use of plasma genotyping in cancer is illustrated in Figure 1.

### Non-small cell lung cancer

The increasing number of “targetable” genotypes in NSCLC and understanding of tumor resistance to targeted therapies has led to rapid, non-invasive, longitudinal assays to repeatedly assess tumor biology throughout treatment.

### ctDNA for NSCLC genotyping in advanced-stage NSCLC

The combination of more targetable genotypes and minimally invasive diagnostic tools (e. g. endobronchial ultrasound) that result in small specimens [4–6] has led to the development of alternative, noninvasive testing methods, such as the U. S. Food and Drug Administration (FDA)-approved targeted ctDNA assay (Cobas) for *EGFR* genotyping or the CLIA (Clinical Laboratory Improvement Amendments)-certified plasma droplet digital polymerase chain reaction (ddPCR) assay. ddPCR is a highly sensitive (*EGFR* exon 19 deletion, 82%; *EGFR* L858R mutation, 74%) and quantitative approach that allows for the longitudinal monitoring of treatment response [7, 8]. Although the specificity of these PCR-based platforms allows for the initiation of EGFR-targeted therapy on the basis of positive plasma testing, negative results must be confirmed by tumor tissue genotyping [9]. While most clinically validated assays are focused on a single predefined gene, next-generation sequencing (NGS) of ctDNA can broadly interrogate the tumor molecular profile across a range of genes and variant types. Hybrid capture-based NGS platforms have already been evaluated in NSCLC [10–12]. Overall, 75% of patients with NSCLC harbor potentially actionable genomic aberrations in ctDNA, although concordance with tissue is suboptimal (specificity, 63.5%) [11, 13–15].

Tumor NGS can help monitor tumor dynamics and detect acquired *ALK* resistance mutations in crizotinib-resistant patients [14]. Our group has studied various NGS methods and found favorable diagnostic accuracy using a bias-corrected targeted ctDNA NGS (2/3 *ALK*; 2/3 *RET*; 2/2 *ROS1*) [16] or using amplicon-based sequencing (6/7 *ALK*; 2/2 *ROS1*) [17]. A larger prospective study is needed to determine the most reliable method for identifying targetable fusions in ctDNA.

At disease progression, ctDNA constitutes a promising alternative to tissue biopsies and is a well-established approach in the *EGFR* setting. Plasma genotyping is widely used as a screening test for detection of the EGFR T790M resistance mutation, with tumor biopsy needed only if the result is negative [1, 17, 18]. It remains unknown, however, if treatment should be adjusted on the basis of isolated plasma variations. Ongoing trials, such as the (APPLE)-EORTC study [19], will help determine the value of ctDNA analysis in treatment selection.

Clinical trials that validated the use of plasma NGS to guide therapy have demonstrated encouraging results [20–22]. In 323 patients with NSCLC, the addition of ctDNA analysis to tissue NGS analysis increased the identification of driver alterations and resulted in an 85.7% rate of objective response or stable disease [20].

### Screening and minimal residual disease in early-stage NSCLC

The National Lung Screening Trial [23] and the Dutch-Belgian Randomized Lung Cancer Screening Trial (NELSON) [24] demonstrated that low-dose computed tomography (CT) screening reduces the mortality rate in lung cancer. Benign lung nodules (false positives) generate invasive procedures. Deep ctDNA sequencing is a more specific and potentially complementary approach to low-dose CT screening in lung cancer but is limited by the low or absent DNA shed of early-stage tumors [25, 26]. Combining ctDNA with other circulating biomarkers such as transcriptomics (ctRNA) could improve sensitivity, while white blood cell sequencing to eliminate “false-positive” variants linked to clonal hematopoiesis may increase specificity [27–29].

ctDNA analysis is also used to detect minimal residual disease (MRD) with plasma genotyping. Tumor molecular profiles from previous biopsies are used to build personalized PCR-based assays with improved sensitivity and specificity. In the TRACERx study, ctDNA from 100 patients was analyzed at the time of diagnosis and followed after definitive treatment. Patient-specific multiplex PCR assays (threshold: ≥2 variants for positivity) demonstrated high sensitivity (93%) and specificity for MRD detection [26, 30].

In another study, ctDNA was analyzed using cancer personalized profiling by deep sequencing (CAPP-seq) in 255 samples from 40 patients with stage I–III lung cancer (stage II–III, *n* = 33) treated with curative intent and 54 healthy adults. It found that in 94% of patients with stage I–III lung cancer with disease recurrence, ctDNA was detectable in the first post-treatment blood sample and preceded radiographic progression by 5.2 months (median), indicating that detection of post-treatment ctDNA should prompt treatment tailored to the patient’s ctDNA analysis to prevent disease progression [31].

### Gastrointestinal tract cancers

#### Esophageal carcinoma

ctDNA analysis of patients with squamous cell carcinoma (SCC) of the esophagus with the use of a 90-gene panel was associated with sensitivity of 94% and 75%, respectively, when detecting ≥1 or ≥2 mutant genes, suggesting that ctDNA analysis can help monitor treatment effect in these patients [32]. In multivariate analysis, a higher plasma cyclin D1 (*CCND1*; 11q13) to dopamine receptor D2 (*DRD2*; 11q22-23) ratio (C/D ratio) was significantly correlated with worse prognosis [33]. In another study, high concordance of multiple somatic mutations was found between plasma ctDNA and tumor (primary or metastases) DNA (83%–100%). The allele frequencies of the mutations increased with tumor burden and preceded radiologic evidence of tumor recurrence by 6 months [34].

In 29 patients with localized esophageal carcinoma treated with chemoradiotherapy, baseline ctDNA levels (NGS-based CAPP-seq) were correlated with metabolic tumor volume and squamous histology. The 2-year overall survival (OS) rates for pretreatment ctDNA-positive vs. ctDNA-negative patients were 47% and 86%, respectively (HR = 6.0; *p* < 0.05). Compared to patients with undetectable ctDNA, detection of ctDNA post-treatment was predictive of shorter event-free survival (EFS, *p* < 0.0001) and shorter time to distant metastasis (*p* < 0.0001) [35].

#### Gastric cancer

ctDNA analysis is used to detect and monitor HER2 copy numbers in patients with gastric cancer [36]. The preoperative ratio of HER2 to RPPH1 (ddPCR) has been correlated with tumor HER2 status (*p* < 0.001; sensitivity, 0.73; specificity, 0.93) [37].

In another study, the concordance rate of HER2 amplification detected between formalin-fixed, paraffin-embedded (FFPE) samples vs. ddPCR and immunohistochemical (IHC) analysis/fluorescence *in situ* hybridization (FISH) was 92%. The concordance rate of FFPE with ctDNA was 62.5%. HER2 positivity by ctDNA analysis was associated with significantly shorter OS compared to HER2 negativity [38]. In late-stage gastric cancer, ctDNA mutations were associated with poor prognosis [39].

In a multiple-parallel cohort, ctDNA-guided plasma-based digital sequencing in patients with metastatic solid tumors, including gastric cancer, identified somatic alterations in 78% of the 76 patients with gastric cancer, and 33% of these 76 patients had targetable alterations. Ten patients with gastric cancer received molecularly matched therapy, which resulted in response and disease control rates of 67% and 100%, respectively [40].

#### Colorectal cancer

In a prospective study of 106 patient samples of CRC, ctDNA analysis of *KRAS* and *BRAF* mutational status was compared to the analysis of tumor tissue [2]. The specificity and sensitivity of ctDNA were both 100% for *BRAF V600E* mutation and were 98% and 92%, respectively, for *KRAS* point mutations. Overall, ctDNA was detected in 100% of patients with metastatic CRC, suggesting that ctDNA analysis could replace tumor analysis in these patients [2].

ctDNA has been used to detect MRD in the adjuvant setting in stage II CRC [41, 42]. In a prospective study of 230 patients, [43] the recurrence rate among patients who did not receive adjuvant chemotherapy was 79% (11/14) in patients with detectable ctDNA vs. 9.8% (16/164) in patients with undetectable ctDNA (HR = 18; *p* < 0.001). ctDNA detection after completion of chemotherapy was associated with shorter recurrence-free survival (HR = 11; *p* = 0.001) suggesting the presence of MRD [43].

The same investigators analyzed ctDNA in patients with stage III CRC post-operatively, during, and after adjuvant chemotherapy [44]. In 95 patients who received adjuvant chemotherapy, serial ctDNA analysis identified residual metastatic disease not evident on imaging studies [44].

In another longitudinal cohort study, ctDNA was used to monitor tumor burden in 21 CRC [45] patients with localized disease who had ctDNA analysis 3 months post-operatively; disease recurrence within 3 years occurred in all 6 patients with detectable ctDNA at 3 months compared to 27% (4/15) of those with undetectable ctDNA at 3 months (HR = 37.7; *p* < 0.001). Of 18 patients who had surgical resection of liver metastases, ctDNA detection 3 months post-surgically predicted disease recurrence compared to patients with undetectable ctDNA and metastatic CRC (HR = 4.9; *p* = 0.007). Therefore, postoperative ctDNA detection identified patients at high risk of relapse [45].

#### Pancreatic and biliary tract carcinomas

The role of ctDNA is particularly important in patients with pancreatic and biliary tract carcinomas because biopsy samples are often inadequate for molecular profiling. In a prospective analysis in 26 patients with pancreatic (*n* = 18) or biliary (*n* = 8) cancer, tumor sequencing using a 54-gene panel failed in 9 patients (35%), and of the remaining 17 patients, 90.3% of the mutations detected in the tumor biopsies were also detected using ctDNA. The diagnostic accuracy of ctDNA sequencing was 97.7% (5 informative genes: sensitivity, 92.3%; specificity, 100%) [46].

In pancreatic cancer, high ctDNA levels of KRAS and/or other mutations have been associated with poor progression-free survival (PFS) and/or OS [47, 48]. However, low concordance between the blood and tissue samples has been reported in patients with pancreatic ductal adenocarcinoma [49].

In a prospective study in advanced pancreatic cancer, ctDNA was analyzed for *KRAS* mutations using blood samples collected prior to gemcitabine or FOLFIRINOX (5-fluorouracil, oxaliplatin, irinotecan, and leucovorin) treatment and monthly during treatment (median follow-up, 3.7 months). Disease progression was more frequent in patients with ctDNA-identified *KRAS* mutations at baseline compared to *KRAS*-mutant-negative patients (9/10 vs. 1/4; *p* = 0.01). The baseline ctDNA level was associated with PFS (*p* = 0.014) and OS (*p* = 0.01). ctDNA level changes were correlated with imaging studies and CA19-9 levels, indicating that ctDNA may be used to monitor disease activity in these patients [47]. In other studies, ctDNA analysis detected mutations in up to 48% of patients, and these mutations were associated with shorter OS. In another study, ctDNA analysis (ddPCR for rare *KRAS* mutations) detected mutations in 31% of 105 patients with pancreatic ductal adenocarcinoma who underwent pancreatoduodenectomy. Detectable ctDNA KRAS mutations were associated with shorter OS compared to patients without mutations (13.6 months vs. 27.6 months; *p* < 0.0001) [48]. Other investigators using ctDNA analysis detected *KRAS* among other mutations (*TP53*, *SMAD4*, *STK11*, *PIK3CA*, *NRAS*) using ctDNA in 48% of patients with advanced pancreatic adenocarcinoma. These mutations were strongly correlated with shorter OS compared to OS of patients with undetectable mutations (6.5 vs. 19.0 months; *p* < 0.001) [50].

ctDNA analysis is a promising prognostic marker in early-stage pancreatic cancer that could also help guide treatment after Whipple surgery [51]. ctDNA analysis of pre- and post-operative plasma samples and tumor tissue (*n* = 42) using PCR-based SafeSeqS assays to identify *KRAS* mutation (codons 12, 13, and 61) identified RAS mutations in 90.5% of tumor samples and in 62.2% of 37 pre-operative and 37.1% of 35 post-operative plasma samples. ctDNA detection was an independent factor predicting shorter recurrence-free survival compared to undetectable ctDNA preoperatively (10.3 months vs. not reached, HR = 3.4; *p* = 0.005) and postoperatively (5.4 months vs. 17.1 months, HR = 5.4; *p* < 0.0001) [51].

In ctDNA promoter hypermethylation analysis of a selected gene panel, the number of methylated genes was significantly higher in patients with pancreatic adenocarcinoma than in healthy individuals or patients with acute pancreatitis (mean, 8.41 vs. 4.74, respectively; *p* < 0.001), suggesting that ctDNA promoter hypermethylation may be used for diagnostic purposes in pancreatic adenocarcinoma [52].

ctDNA analysis can also inform the diagnosis, prognosis, and treatment of patients with biliary tract cancer. In patients with metastatic locally advanced/metastatic biliary tract cancer, the concordance rate between plasma ctDNA analysis and tumor tissue analysis (15-gene panel) was 74%; this rate was 92% in intrahepatic tumors. ctDNA variant allele frequency (VAF) was significantly correlated with tumor load and PFS, and the mutational profile changed after chemotherapy in 36% of patients [53].

#### Hepatocellular carcinoma

ctDNA analysis has been investigated in hepatocellular carcinoma (HCC) [54–56]. In patients who underwent hepatectomy or liver transplantation, disease recurrence and extrahepatic metastases were more frequent in those with mutations detected by ctDNA analysis vs. others (*p* = 0.01 and 0.04, respectively) and ctDNA was an independent factor predicting invasion of the portal vein (odds ratio = 6.10) [54]. In other studies, hotspot mutations in the *TERT*, *CTNNB1*, and *TP53* genes detected in the plasma of patients with HCC were associated with vascular invasion and likely predicted a shorter recurrence-free survival time [55].

#### Breast cancer

The concordance between ctDNA and tissue DNA in breast cancer varies by stage and subtype [57]. In patients with early-stage breast cancer, *PIK3CA* mutations have been identified presurgically in ctDNA with high sensitivity (93.3%) and specificity (100%) [58]. In a retrospective analysis, the concordance between tissue DNA and ctDNA (digital sequencing of plasma-derived DNA) was robust in *PIK3CA* mutation and ERBB2 amplification analyses (Cohen’s κ = 0.64 and 0.77, respectively) but poor in *TP53* mutation and EGFR amplification analyses (Cohen’s κ = 0.18 and 0.33, respectively) [59, 60]. In another study, *TP53* and *PIK3CA* mutant allele frequencies were associated with response to therapy and PFS [60]. When PCR and targeted exome sequencing were used to detect the hotspot *AKT1* E17K mutation in two cohorts of patients with advanced metastatic breast cancer (MBC), the concordance rates between tissue and blood samples were 98% and 97.1% [61].

ctDNA analysis has been used extensively in large trials in hormone-receptor–positive, HER2-negative advanced breast cancer. In the BOLERO-2 study, *PIK3CA* mutations by ctDNA analysis were associated with efficacy of everolimus [62, 63]. In the BELLE-3 study, patients progressing on or after mTOR inhibition and endocrine therapy [64] with *PIK3CA* mutations (detected by ctDNA or tissue DNA analysis; concordance, 80%) had significantly longer PFS in the buparlisib plus fulvestrant arm compared to the fulvestrant arm [64, 65].

In patients with HER2-positive breast cancer treated with an anti-HER1/HER2 tyrosine kinase inhibitor, ctDNA analysis for HER2 amplification was associated with disease progression (4/6, 66.7%), whereas *TP53* mutations (3/6, 50%) and PI3K/mTOR pathway alterations (3/6, 50.0%) were associated with disease resistance. Dynamic ctDNA analysis identified drug resistance with sensitivity 85.7% and specificity of 55.0%; and had a high concordance rate with CT imaging studies (82.1%) [66].

In patients with non-metastatic triple-negative breast cancer, ddPCR for customized ctDNA analysis detected alterations in 75% of patients at baseline. Lower ctDNA levels were associated with longer OS during neoadjuvant chemotherapy [67]. In this setting, targeted MRD sequencing using serial ctDNA monitoring predicted tumor recurrence 7.9 months (median) before clinical evidence of relapse [68].

Other investigators found that a panel of cell-free methylation markers was a strong predictor of OS in MBC and may have clinical usefulness in risk disease monitoring [69]. Methylated ctDNA from the promoter region of *RASSF1A* was more sensitive than carcinoembryonic antigen and CA15-3 for monitoring response to neoadjuvant chemotherapy [70]. ctDNA analysis of a 6-gene methylation panel for diagnosis of breast cancer had sensitivity of 79.6% compared to healthy individuals and 82.4% compared to benign disease control (specificity, 72.4% and 78.1%, respectively). This test complemented mammography or ultrasonography [71].

ctDNA for assessment of resistance to aromatase inhibitors as first-line therapy was also prospectively studied in MBC (*n* = 83) [72]. *ESR1* mutations were detected in 56.4% (22/39) of patients who had disease progression 6.7 months (median) prior to clinical progression [72]. On the basis of detection of *ESR1* mutations in primary breast cancer using ctDNA analysis at a very low allele frequency, in contrast to a high allele frequency in metastases, it is plausible that in some tumors rare *ESR1*-mutant clones may be enriched by endocrine therapy [73].

#### Gynecologic cancers

ctDNA analysis has contributed to diagnosis and monitoring of patients with gynecologic cancers. High ctDNA levels have been associated with poor PFS and OS in patients with resistant epithelial ovarian cancer treated with bevacizumab (*n* = 144) [74]. In patients with high-grade serous ovarian carcinoma who received standard-of-care therapy, p53 alterations identified using ctDNA analysis at baseline were correlated with volume of disease, and a decrease in *TP53* mutant allele fraction ≤60% after 1 cycle of chemotherapy was associated with shorter time to disease progression [75].

In patients with gynecologic cancer (*n* = 44), ctDNA analysis using ddPCR detected alterations in 93.8% of patients, and detected cancer in 6 patients, 7 months prior to radiologic evidence on CT imaging studies [76]. ctDNA levels were correlated with serum CA-125 levels and CT imaging studies, and they were an independent factor predicting OS [76].

Plasma DNA analysis for detection of chromosomal instability using copy-number alterations in patients with an adnexal mass (*n* = 68) and in healthy individuals (*n* = 44) improved detection of malignancy (AUC = 0.89) compared to serum CA-125 (AUC = 0.78) or the RMI (risk of malignancy) index (composite of serum CA 125 level, ultrasound scan result and menopausal status) (AUC = 0.81) [77].

Data suggest that human papillomavirus (HPV) detection by ctDNA analysis is a surrogate marker for HPV-associated cervical cancer and can guide antiviral therapy. In patients with HPV 16/18-associated cervical cancer and tumors >2 cm at diagnosis, HPV ctDNA was identified in 11 of 13 patients and levels were associated with tumor dynamics [78]. In another study, HPV ctDNA was detected in all patients (*n* = 19) with HPV-positive metastatic cervical cancer but in 0 healthy blood donors (*n* = 45) [79]. Of 9 patients who received tumor-infiltrating lymphocyte (TIL) immunotherapy, the HPV genotype of the patients’ tumors was identified in serum samples from all patients, and 2 patients with complete response had persistent clearance of HPV ctDNA [79].

### Genitourinary cancers

#### Prostate cancer

In patients with metastatic castration-resistant prostate cancer (mCRPC), ctDNA analysis can identify molecular alterations that are associated with clinical outcomes and can guide therapy. Detection of biallelic *BRCA2* gene loss by ctDNA analysis indicated that patients may benefit from therapies targeting defective DNA repair [80]. Others identified alterations in all analyzed patients with mCRPC treated with enzalutamide, including alterations in DNA damage repair and PI3K pathway genes [81]. Aberrations in the androgen receptor (*AR*) gene by ctDNA analysis have been correlated with resistance to enzalutamide and abiraterone treatment and *AR* amplification was more common in patients whose disease progressed on enzalutamide compared to abiraterone or other agents (*p* = 0.02) [82]. Targeted NGS covering all AR coding bases using plasma from patients treated with abiraterone (control, patients’ normal circulating DNA) identified AR copy numbers in 82.5% (80/97) of patients and demonstrated that 45% of tumors had AR gain or T878A or L702H changes before abiraterone treatment, which were associated with shorter PFS and OS [83]. Other investigators also demonstrated that circulating AR copy number gain is a useful biomarker [84]. In patients with CRPC treated with docetaxel followed by enzalutamide (*n* = 59), patients with AR copy number gain (36%) had higher levels of PSA, alkaline phosphatase, and lactate dehydrogenase (LDH) and shorter PFS (*p* = 0.0004) and OS (*p* = 0.0003) compared to those without AR copy number gain. In multivariate analysis, a decrease in PSA ≥50% and AR copy number gain were associated with longer PFS and OS [84].

In a retrospective study, ctDNA genomic profiling using Guardant 360™ demonstrated ≥1 alteration in 94% of 514 men with progressive mCRPC, and had good concordance with the correspondent tumor tissue [85]. Higher numbers of ctDNA alterations were associated with shorter time to treatment failure (HR = 1.05, *p* = 0.026) in patients treated with chemotherapy or androgen inhibitors [85].

#### Renal cell carcinoma

Total serum ctDNA levels and CpG island methylation of *RASSF1A* and *VHL* were shown to support the diagnosis of renal cell carcinoma (RCC) and VHL methylation indicates clear cell RCC [86]. Higher levels of ctDNA were identified in metastatic RCC or necrotic RCC compared to benign tumors, and they were associated with poorer DFS [86]. Using Guardant 360 ctDNA analysis, 78.6% of 220 patients with RCC had >1 alteration and the most frequent alterations were TP53 (35%), VHL (23%), EGFR (17%), NF1 (16%), and ARID1A (12%) [87]. Higher rates of detection after systemic therapy compared with baseline were noted for *NF1* (21% vs. 3%), *TP53* (49% vs. 24%), and *VHL* (29% vs. 18%), indicating clonal evolution of genomic alterations [87].

#### Bladder cancer

In patients with advanced urothelial carcinoma, high rates (86%–90%) of aberrations using Guardant 360 ctDNA analysis were found; the most common aberrations were *TP53* (48%), *ARID1A* (17%), and *PIK3CA* (14%) [88, 89]. Using a 62-gene panel (FoundationACT), 73% of patients with metastatic urothelial cancer were found to have ≥1 aberration and the most frequent alterations were *TP53* (68%) and *TERT*-promoter (38%) [90]. In another study, 36% (129/363) of patients with non-muscle-invasive bladder cancer and 11% (44/403) of patients with muscle-invasive bladder cancer who underwent radical cystectomy had ≥1 *FGFR3* or *PIK3CA* mutations and high ctDNA levels were associated with disease progression. ctDNA levels in the urine and plasma were positively correlated and indicated that higher levels of *FGFR3-* and *PIK3CA*-mutated DNA can predict disease progression [91].

#### Melanoma

In patients with metastatic melanoma, identification of *BRAF* mutations in ctDNA has been associated with higher disease burden and worse prognosis and may precede clinical evidence of disease progression. In patients with unresectable advanced-stage metastatic melanoma, *BRAF* and *NRAS* mutations identified in ctDNA analysis at baseline and during treatment with targeted therapy against BRAF or immunotherapy have been associated with larger tumors, increased LDH levels, and brain metastases [92]. Other investigators found that in this setting, the presence of BRAF mutations in ctDNA analysis was associated with a higher number of metastatic sites, higher serum LDH levels or S100 protein concentration, and shorter OS [93].

In one study, 73% of 48 patients with metastatic melanoma had tumor-associated *BRAF* and *NRAS* alterations in ctDNA analysis [94]. In another study, patients with the BRAF V600E mutation in ctDNA had shorter PFS and OS compared to those without the mutation (*p* = 0.02 and *p* = 0.017, respectively) [95]. Others also demonstrated that lower baseline ctDNA levels were associated with higher rates of response and PFS [94]. In patients with BRAF V600E/V600K-positive tumors enrolled in 4 different studies of dabrafenib or trametinib, ctDNA analysis identified BRAF V600E and BRAF V600K mutations in 76% and 81% of 732 patients, respectively. Patients with undetectable ctDNA *BRAF* mutations at baseline had higher rates of response, PFS, and OS than those with ctDNA BRAF mutations [96]. Circulating BRAF mutations have been identified in some patients prior to clinical evidence of disease progression [97]. In patients with melanoma who received adoptive transfer of activated autologous TILs, ctDNA analysis for BRAF V600E (*n* = 48, 388 serum samples) demonstrated a strong correlation between an early peak of circulating V600E mutation and objective response. Patients whose serum had an early ctDNA peak followed by undetectable ctDNA had a higher likelihood of having a complete response in 1–2 years [97]. Others also found a correlation between serial ctDNA analysis of *BRAF* and *NRAS* status and tumor response. PFS was longer in patients with an early decrease (1–4 weeks post-treatment) in ctDNA levels than in patients with unchanged or increased ctDNA levels post-treatment (HR = 2.6; *p* = 0.05) [98]. In the post-surgical setting, ctDNA analysis of *BRAF* and *NRAS* mutations predicted OS in 161 patients with high-risk stage II/III melanoma who underwent surgical resection followed by adjuvant bevacizumab for 1 year [99]. The 5-year OS rates were 33% and 65% for patients with detectable and undetectable ctDNA levels, respectively. After adjustment for performance status, patients with detectable ctDNA had shorter OS compared to those with undetectable ctDNA (HR = 2.63; *p* = 0.003) [99].

An intriguing application of longitudinal ctDNA analysis is the distinction of pseudoprogression from true progression [100]. ctDNA for BRAF and NRAS mutations was analyzed at baseline and at 12 weeks of treatment with PD-1 antibodies with or without ipilimumab [100]. Overall, 23.2% (29/125) of patients had initial disease progression by imaging studies. Thirty-one percent (9/29) of patients had pseudoprogression and 69% (20/29) had true progression. The 9 patients with confirmed pseudoprogression had undetectable ctDNA at baseline or detectable ctDNA at baseline followed by >10-fold decrease (favorable ctDNA profile). Eighteen of 20 patients with true progression had detectable ctDNA at baseline that remained stable or increased (unfavorable ctDNA profile). Among patients with confirmed true progression, the 1-year OS rates were 82% and 39% in those with favorable ctDNA and unfavorable ctDNA profiles, respectively (HR = 4.8; *p* = 0.02) [100].

### Sarcoma

#### Ewing sarcoma

In patients with localized or metastatic Ewing sarcoma (EWS), copy numbers of the EWSR1 fusion sequence in plasma were associated with tumor volume. Rapid decrease in ctDNA levels of EWSR1 was noted during initial chemotherapy, and increase in ctDNA levels indicated disease recurrence [101]. As a driving EWS-ETS translocation specific to each tumor is identified in up to 95% of patients with Ewing sarcoma, investigators used long-range PCR analysis to identify tumor-specific EWS-ETS breakpoints in plasma DNA [102]. In children with metastatic Ewing sarcoma and primary localized osteosarcoma, detection of ctDNA was associated with inferior outcomes [103]. In patients with localized EWS and detectable ctDNA, the 3-year rates of EFS and OS were lower compared to those with undetectable ctDNA (*p* = 0.006 and *p* = .01, respectively). The respective rates in localized osteosarcoma for EFS were 48.6% vs. 82.1% (*p* = 0.006) and for OS were 79.8% vs. 92.6% (*p* = 0.01); the risk of death increased proportionately with ctDNA levels [103].

#### Gastrointestinal stromal tumors

In patients with gastrointestinal stromal tumors (GIST), ctDNA harboring *CKIT* or *PDGFRA* was used as a tumor-specific biomarker and the amount of mutant-free circulating DNA was correlated with disease course [104]. In patients with TKI-refractory GIST treated with dovitinib, genotyping of the KIT gene in exon 17 of serum ctDNA using beads, emulsions, amplification, and magnetics assays identified mutations associated with disease resistance [105]. Other investigators suggested that detection of secondary *C-KIT* mutations in ctDNA may improve the selection of targeted agents [106].

#### Soft tissue sarcoma

In patients with metastatic soft tissue sarcoma, ctDNA was detected in 36% (4/11) of patients and TP53/PIK3CA mutations in ctDNA analysis were concordant with the primary tumor in 2 of 4 patients [107].

#### Brain tumors

In brain tumors, ctDNA analysis is used as a non-invasive alternative to tumor biopsies that are associated with significant risk. In patients with glioblastoma multiforme (GBM), ctDNA analysis identified the EGFRvIII deletion in 3 of 13 patients, which was correlated with tumor tissue analysis [108]. This mutation is identified in approximately one third of patients with GBM and is associated with resistance to chemotherapy and radiotherapy. ctDNA levels were correlated with the extent of tumor resection. This test may help select patients for anti-EGFRvIII therapy and monitor response to treatment [108]. In patients with neuroblastoma, serum MYCN amplification (real-time quantitative PCR, sensitivity 86%, specificity 95% compared with tissue analysis) was associated with OS, suggesting that it may help select treatment prior to tumor biopsy, particularly for patients younger than 18 months whose risk assessment and treatment depend on MYCN amplification status [109]. In patients with glioma, the *IDH1* R132H mutation was identified in plasma with a specificity of 100% and sensitivity related to the tumor volume and contrast enhancement, suggesting that it may help in the diagnosis of patients not amenable to biopsy [110].

### Lymphoma

#### Classical Hodgkin lymphoma

In patients with classical Hodgkin lymphoma, an *XPO1* mutation detected using ctDNA analysis at the end of treatment was associated with a tendency toward shorter PFS compared to patients without the mutation, suggesting that plasma ctDNA may be clinically useful for the noninvasive management of this disease [111].

#### Diffuse large B-cell lymphoma

In patients with relapsed, refractory *de novo*, or transformed diffuse large B-cell lymphoma (DLBCL) treated with panobinostat with or without rituximab, ctDNA was detected in ≥1 plasma sample in 96% of the patients and its increase was associated with resistance to treatment [112]. In another study of patients with lymphoma and healthy subjects, the amount of ctDNA at diagnosis was strongly correlated with clinical indices and was independently predictive of patient outcomes [113]. ctDNA genotyping could help distinguish indolent follicular lymphomas from those that transformed into DLBCL and classify transcriptionally defined tumor subtypes, including DLBCL cell of origin [113].

Other investigators used immunoglobulin high-throughput sequencing (Ig-HTS) [114] to analyze circulating leukocytes and ctDNA of patients with DLBCL. At baseline, Ig clonal rearrangement was detected in 82% and 71% of patients in ctDNA and circulating cells, respectively (*p* = 0.68); at relapse, the respective rates were 100% and 30% (*p* = 0.001). Interestingly, Ig-HTS detection preceded radiologic evidence of recurrent disease by 88 days with high sensitivity and specificity, indicating that it may be a surrogate marker for monitoring disease after complete remission is achieved [114].

In a retrospective study in patients with DLBCL after first-line treatment, disease progression was evident on imaging studies a median of 3.5 months (range, 0-200 months) after detection on ctDNA analysis of the clonal Ig gene sequence [115].

In contrast to these data showing that ctDNA is associated with prognosis and can be identified prior to radiologic evidence of recurrent DLBCL, in patients with newly diagnosed primary central nervous system lymphoma who had 34-gene panel high-throughput sequencing of primary tumors followed by targeted sequencing of identified somatic mutations on plasma, the correlation between ctDNA concentration and tumor volume was insignificant (R^2^ coefficient = 0.01) [116]. Overall, 88% of patients with MYD88 L265P in tissue had an identifiable L265P variant in their ctDNA and OS was not significantly correlated with mutations detected in ctDNA [116].

#### Clinical trials across tumor types

In patients with advanced cancer, circulating nucleic acid biomarker analyses had promising clinically important multipurpose utility awaiting further studies [117]. In an analysis of 105 patients using the Sequenom MassArray System and OncoCarta panel for somatic mutations, the ctDNA concentration was 3 times higher in patients with advanced cancer compared to healthy volunteers. Although the concordance between matched ctDNA and archival tumor tissue was high for selected ‘hot-spot’ mutations (*KRAS*, *BRAF*, *PIK3CA*), some differences were noted between archival tumor and ctDNA. Factors predicting longer OS in multivariate analysis were lower ctDNA concentration, higher albumin levels, and better performance status [117].

We have previously published our experience analyzing ctDNA and archival primary or metastatic tumor tissue (FFPE) from patients with advanced metastatic cancer who were referred to our Phase I program at MD Anderson for participation in clinical trials [118]. We found that the concordance rates between mutations in archival tissue and ctDNA were high in patients with refractory cancer types that progressed on systemic therapy. These rates were 91%, 99%, 83%, and 91% for BRAF, EGFR, KRAS, and PIK3CA mutations, respectively [118]. Patients with >1% of KRAS mutant ctDNA had shorter OS compared to those with ≤1% of KRAS mutant ctDNA (4.8 vs. 7.3 months, *p* = 0.008). Patients with >1% of mutant ctDNA (BRAF, EGFR, KRAS, or PIK3CA) had shorter OS compared to those with ≤1% of mutant ctDNA (5.5 vs. 9.8 months, *p* = 0.001) [118].

Other investigators demonstrated that ctDNA and circulating tumor cells are distinct biomarkers, as ctDNA was also detected in patients without any evident circulating tumor cells [119]. ctDNA analysis demonstrated high detection rates (>75%) in advanced breast, bladder, colorectal, gastroesophageal, hepatocellular, head and neck, melanoma, ovarian, and pancreatic cancer. Low detection rates (<50%) were noted in primary brain, renal, prostate, and thyroid cancers. In localized tumor stage, the rate of ctDNA detection was 73% in CRC, 57% in gastroesophageal, and 50% in breast adenocarcinoma. In metastatic CRC (*n* = 206), ctDNA analysis for KRAS mutation was associated with high sensitivity (87.2%) and specificity (99.2%). ctDNA analysis to assess resistance mechanisms to anti-EGFR treatment in patients with CRC demonstrated that 96% (23/24) of patients developed ≥1 mutation in genes involved in the mitogen-activated protein kinase pathway [119].

In a study of clonal evolution, exome sequencing of ctDNA using serial plasma from 6 patients with breast, ovarian, or lung cancer demonstrated that emergence of resistance was associated with increased mutant allele fractions, and an activating *PIK3CA* mutation was noted after treatment with paclitaxel [120]. In another study with ctDNA analysis of 23 patients with various tumor types treated with PI3K-AKT-mTOR pathway or MEK inhibitors, clonal response to treatment was noted and some clones changed over time discordantly. Increasing mutational levels were associated with poorer prognosis [121].

Other investigators used NGS to analyze 54 genes and copy number variants in three genes (EGFR, ERBB2 and MET) on ctDNA of patients with various tumor types [122]. Overall, 171 patients with lung (*n* = 39), breast (*n* = 39), glioblastoma (*n* = 33), or other cancers were analyzed. Actionable mutations were noted in 40% (TP53 29.8%, EGFR 17.5%, MET 10.5%, PIK3CA 7%, and NOTCH1 5.8%). Eighteen percent (6/33) of patients with glioblastoma had actionable mutations [122]. The same investigators analyzed 168 patients with diverse cancers [123]. In ctDNA analysis, 58% of patients had ≥1 alteration. Among them, 71.4% had ≥1 alteration potentially targeted by an FDA-approved drug. The concordance rates between ctDNA analysis and tissue analysis were: TP53 70.3%, EGFR 70.3%, PIK3CA 88.1%, and ERBB2, 93.1%. In patients with ≥1 alteration, those with ctDNA ≥5% of total DNA had shorter OS compared to patients with ctDNA <5% (median, 4.03 months vs. NR, *p* < .001) [123]. With the use of checkpoint inhibitor–based immunotherapy across various histologies, correlation between high alteration number detected in blood-derived ctDNA and favorable response, PFS and OS has been reported [124].

In a meta-analysis of patients with solid tumors (39 studies, 4,052 patients), detection of ctDNA in plasma was associated with shorter OS in multivariable analyses [HR, 2.70; *P* < .001) compared to patients with undetectable ctDNA [125].

Selected studies by tumor type, the gene (s) used for ctDNA analysis, and outcomes are summarized in Table 1.

**Table 1:** Selected studies by tumor type, the gene(s) used for ctDNA analysis, and outcomes

Year, First Author [Ref]	Study Type	Tumor Type	Number of Patients	Biomarker Method	Tested Genes	Outcome
**Lung cancer**						
2016, Adrian G. Sacher [8]	Prospective	Advanced NSCLC	180	ddPCR	*KRAS, EGFR*	Detection of KRAS and EGFR mutations, lower turnaround time compared to tissue
2016, Jeffrey C. Thompson [10]	Cohort	Advanced NSCLC	102	Hybrid capture NGS	70 cancer-related genes	Detection of potentially actionable variants in 75% of patients; concordance with tissue, 79%
2018, Nicolas Guibert [13]	Cohort, blinded to tissue	Advanced NSCLC	168 specimens from 46 patients	Amplicon-based NGS	36 cancer-related genes	Detection of EGFR mutations, rare variants and fusions with high specificity. Early detection of resistance mechanisms in serial samples.
2018, Charu Aggarwal [20]	Prospective	Advanced NSCLC	323	Hybrid capture	73 cancer-related genes	Detection of actionable alterations in 20% of stage IV M1b patients in plasma but not tissue. Complementarity of tissue and plasma
2014, Aaron M. Newman [25]	Cohort	Early-stage lung cancer	103	Hybrid capture	16 cancer-related genes + 8 proteins	Detection of ctDNA in early stages (stage I sensitivity, 50%)
2017, Christopher Abbosh [26]	Cohorts	Early-stage lung cancer	96	Patient-specific multiplex PCR	10–22 SNVs	MRD and subclonal evolution
**Gastro-intestinal tumors**						
2016, Honglei Luo [32]	Cohort	Esophageal, SCC	8	Illumina TruSight sequencing	90 cancer-related genes	Multigene panel has a role in detection and monitoring response to treatment
2010, Hiroki Takeshita [33]	Case-control	Esophageal, SCC	96 patients, 40 controls	PCR-applied biosystems	*CCND1* amplification	Poor prognostic value of CCND1 amplification
2016, Masami Ueda [34]	Cohort	Esophageal, SCC	13	HiSeq2000	53 cancer-related genes	Multigene panel is associated with a greater accuracy of tumor recurrence compared to imaging methods (post-op)
2015, Katsutoshi Shoda [36]	Case-control	Gastric	52 patients, 40 controls	PCR-applied biosystems	*HER-2*	HER2 amplification can be used for therapeutic monitoring
2017, Katsutoshi Shoda [37]	Case-control	Gastric	60 patients, 30 controls	PCR-applied biosystems	*HER-2*	HER2 amplification can be used for therapeutic monitoring
2015, Hideaki Kinugasa [38]	Cohort	Gastric	25	PCR - QX200, Bio-Rad	*HER-2*	High concordance in detection of HER-2 between ddPCR and tissue IHC/FISH
2016, Wen-Liang Fang [39]	Cohort	Gastric	277	TaqMan qPCR	68 mutations (8 genes)	High ctDNA levels are associated with peritoneal recurrence and poor prognosis
2017, Jeanne Tie [43]	Prospective, cohort, multicenter	Colon	230 (1046 plasma samples)	Safe-SeqS PCR	15 cancer-related genes	ctDNA detection after stage II colon cancer resection provides direct evidence of residual disease and identifies patients at very high risk of recurrence.
2018, Jeanne Tie [44]	Prospective, cohort, multicenter	Colon	95	Safe-SeqS PCR	15 cancer-related genes	ctDNA detection after adjuvant chemotherapy for stage III colon cancer resection can identify patients at very high risk of recurrence.
2017, Lone V. Schøler [45]	Longitudinal cohort	Colon	45 (371 plasma samples)	QX200 PCR	Somatic structural variants, *KRAS*	Postoperative ctDNA analysis detects residual disease and identifies patients at very high risk of relapse. Longitudinal surveillance allows early detection of relapse and response to intervention.
2015, Oliver A. Zill [46]	Cohort	Pancreato-biliary carcinomas	26	Illumina Hi-Seq 2500	54 cancer-related genes	ctDNA sequencing is feasible, accurate, and sensitive in identifying tumor-derived mutations.
2016, Kjersti Tjensvoll [47]	Cohort	Pancreatic	14 (53 plasma samples)	Mx3000P rtPCR	*KRAS*	ctDNA can be used as a marker for monitoring treatment efficacy and disease progression.
2016, Naoto Hadano [48]	Cohort	Pancreatic	105	TaqMan assay PCR	*KRAS*	ctDNA can predict poor survival
2017, Daniel Pietrasz [50]	Prospective, cohort	Pancreatic	135	Ion AmpliSeq NGS	112 cancer-related genes	ctDNA is an independent prognostic marker in advanced pancreatic adenocarcinoma
2018, Belinda Lee [51]	Cohort	Pancreatic	42	SafeSeqS assays PCR	*KRAS*	ctDNA analysis is a promising prognostic marker in early-stage pancreatic cancer and guides risk-adaptive treatment strategies. ctDNA detection post-operatively helps to identify disease progression after standard adjuvant chemotherapy.
2016, Stine Dam Henriksen [52]	Prospective case-control	Pancreatic	95 patients, 183 controls	Methylation-specific PCR	28 cancer-related genes	ctDNA promoter hypermethylation is a diagnostic biomarker that helps distinguish malignant from benign pancreatic disease.
2018, Andreas Wolfgang Berger [53]	Cohort	Biliary tract cancer	24	1010× depth Sequencing	15 cancer-related genes	The molecular landscape is represented in ctDNA.
2015, Atsushi Ono [54]	Cohort	Hepatocellular carcinoma	46	Illumina Hi-Seq 2500		ctDNA detection post-surgery reflects tumor progression and disease recurrence.
2016, Wenjun Liao [55]	Cohort	Hepatocellular carcinoma	41	Illumina MiSeq™	Cancer-related genes TERT, TP53, and CTNNB1	ctDNA mutation detection is associated with vascular invasion and predicts a shorter recurrence-free survival time.
2016, Ao Huang [56]	Cohort	Hepatocellular carcinoma	48	QX200 PCR	Cancer-related genes TERT, TP53, and CTNNB1	ctDNA analysis can detect intratumoral heterogeneity and may have a promising role in the therapeutic management.
**Breast cancer**						
2017, Heather A. Parsons [57]	Prospective cohort	Triple-negative breast cancer	26	HiSeq 2500 Illumina	33 cancer-related genes	High concordance between ctDNA analysis and tumor tissue analysis, allowing monitoring of the therapeutic effect.
2014, Julia A. Beaver [58]	Prospective cohort	Breast cancer	29	ddPCR	PIK3CA mutations	In patients with early-stage breast cancer, mutations can be detected in tumor tissue using ddPCR, and ctDNA can be detected in blood before and after surgery.
2016, Diana H. Liang [60]	Retrospective chart review	Breast cancer	100	Illumina Hi-Seq 2500	TP53, PIK3CA, ERBB2, and EGFR genomic alterations	Robust concordance between tissue and blood for detection of PIK3CA mutation and ERBB2 amplification, but not for TP53 mutation and EGFR amplification. Directional changes of TP53 and PIK3CA mutant allele are associated with response to therapy and PFS.
2016, Marion Rudolph [61]	Cohort	Breast cancer	600	HiSeq 2500 Illumina	306 cancer-related genes	AKT1E17K is the most likely disease driver in selected breast cancer patients and its detection in blood is achievable in advanced-stage disease.
2017, Mary Ellen Moynahan [62]	Prospective	Breast cancer	724	QX200 PCR	PIK3CA	Improvement in PFS was maintained using everolimus, irrespective of PIK3CA genotypes (detected by ctDNA), and it was consistent with previous analysis of archival tumor DNA using NGS.
2016, Sarat Chandarlapaty [63]	Prospective	Breast cancer	541	QX200 PCR	ESR1	ESR1 mutations are prevalent in ER-positive metastatic breast cancer treated with aromatase inhibitors. Both Y537S and D538G mutations are associated with aggressive disease biology.
2018, Rosaria Condorelli [65]	Cohort	Breast cancer	3	QX200 PCR	*RB1*	Somatic RB1 mutations can emerge after exposure to CDK4/6 inhibitors.
2016, Fei Ma [66]	Prospective, cohort	Breast cancer	18	HiSeq 2500 Illumina	368 cancer-related genes	ctDNA analysis provides information regarding resistance to treatment and guides administration of anti-HER2 targeted therapy in the metastatic setting.
2017, Francesca Riva [67]	Cohort	Triple-negative breast cancer	46	Illumina MiSeq	*TP53*	ctDNA levels decreased quickly during neoadjuvant chemotherapy (NCT) and helped identify minimal residual disease after surgery. Slow decrease of ctDNA levels during NCT was strongly associated with shorter survival.
2015, Isaac Garcia-Murillas [68]	Prospective, cohort	Breast cancer	55	HiSeq 2500 Illumina	Cancer-related genes	In patients with early stage breast cancer, ctDNA analysis can identify patients at high risk for relapse and guide adjuvant therapy.
2017, Kala Visvanathan [69]	Cohort	Breast cancer	141	cMethDNA assay	10 cancer-related genes	ctDNA gene methylation is a strong predictor of survival outcomes.
2017, Hiroyo Takahashi [70]	Cohort	Breast cancer	87	Methylation-specific PCR	RASSF1A	Met-ctDNA is a more sensitive marker than CEA and CA15-3 and it can be used to monitor clinical tumor response to neoadjuvant chemotherapy.
2016, Ming Shan [71]	Cohort	Breast cancer	749	MethyLight	SFN, P16, hMLH1, HOXD13, PCDHGB7 and RASSF1a	Epigenetic markers in serum have potential for diagnosis of breast cancer.
2018, Charlotte Fribbens [72]	Prospective, cohort	Breast cancer	83	Enhanced tagged-amplicon sequencing (eTAm-Seq)	*ESR1, KRAS, NRAS* and *HRAS*	In patients with progressive disease after first-line aromatase inhibitors, ctDNA analysis demonstrated high levels of genetic heterogeneity and frequent sub-clonal mutations. Sub-clonal KRAS mutations were found at a high frequency.
2016, Peilu Wang [73]	Cohort	Breast cancer	126	Bio-Rad QX100 dd PCR	ESR1	*ESR1* mutations were detected at very low allele frequencies in some primary breast cancers, and at high allele frequency in patients with metastatic breast cancer. *ESR1*-mutant clones are enriched by endocrine therapy.
**Gynecological cancers**						
2014, Karina Dahl Steffensen [74]	Cohort	Ovarian cancer	144	QiaSymphony, multiplex qPCR	ctDNA detection	In patients treated with bevacizumab, high ctDNA levels were associated with poor PFS and OS.
2016, Christine A. Parkinson [75]	Retrospective analysis	Ovarian cancer	40	ddPCR	*TP53*	ctDNA is correlated with volume of disease at the start of treatment.
2015, Elena Pereira [76]	Cohort	Ovarian/ endometrial cancer	44	qPCR using TaqMan^®^, ddPCR	ctDNA detection	ctDNA is an independent predictor of survival in patients with ovarian and endometrial cancers.
2017, Adriaan Vanderstichele [77]	Prospective, cohort	Ovarian cancer	68	HiSeq 2500 Illumina	Chromosome instability	ctDNA analysis demonstrated that chromosomal instability can help detect ovarian cancer.
2012, Maura Campitelli [78]	Cohort	Cervical cancer	16	RT-qPCR	ctDNA detection	ctDNA analysis demonstrated that the HPV mutational insertion is a highly specific molecular marker and it is detected in most patients with stage 2-4 cervical cancer.
2017, Zhigang Kang [79]	Retrospective analysis	Cervical cancer	19	ddPCR	HPV genetic components	HPV genetic insertion in ctDNA represents a promising tumor marker.
**Urological cancers**						
2017, Matti Annala [80]	Prospective, cohort	Prostate cancer	319	NimbleGenSeqCap, Illumina	73 cancer-related genes	Biallelic gene loss detected in ctDNA can help prioritize therapy.
2016, Alexander W. Wyatt [81]	Cohort	Prostate cancer	65	Illumina MiSeq, Ion Ampliseq	19 cancer-related genes	Genomic profiling of ctDNA is feasible in mCRPC patients and provides important insights into enzalutamide response and resistance.
2015, Arun A. Azad [82]	Cohort	Prostate cancer	62	PCR-based BEAMing	AR	AR gene aberrations in ctDNA are associated with resistance to enzalutamide and abiraterone in mCRPC.
2015, Alessandro Romanel [83]	Cohort	Prostate cancer	97	Ion Torrent Sequencing	AR	Plasma AR sequencing can identify primary resistance to abiraterone.
2016, Samanta Salvi [84]	Cohort	Prostate cancer	59	RT-PCR, ddPCR	AR	Detection of circulating AR copy number gain is a non-invasive biomarker for outcome of patients with CRPC treated with enzalutamide.
2017, Sumanta K. Pal [87]	Prospective, cohort	Renal cell carcinoma	220	HiSeq 2500 Illumina	73 cancer-related genes	Higher rates of detection by ctDNA analysis after systemic therapy compared with baseline was noted for *NF1, TP53*, and *VHL*, indicating clonal evolution of genomic alterations.
2018, Neeraj Agarwal [89]	Cohort	Urothelial carcinoma	369	HiSeq 2500 Illumina	73 cancer-related genes	ctDNA NGS identified similar genomic alterations with tumor tissue. The genomic landscape was similar between lower tract and upper tract urothelial carcinoma.
2017, Emil Christensen [91]	Cohort	Urothelial carcinoma	831	ddPCR	FGFR3 and PIK3CA	ctDNA levels in the urine and plasma were positively correlated and indicated that higher levels of *FGFR3-* and *PIK3CA*-mutated DNA can predict disease progression.
**Skin cancer**						
2016, Gregory A. Chang [92]	Cohort	Melanoma	43	ddPCR	*NRAS*, *BRAF*	ctDNA had a higher sensitivity than LDH to detect disease progression.
2016, Anne C. Knol [93]	Cohort	Melanoma	38	RT-PCR	*BRAF*	ctDNA *BRAF* mutation is a prognostic factor of OS and it is correlated with tumor burden.
2015, Elin S. Gray [94]	Cohort	Melanoma	48	ddPCR	*NRAS*, *BRAF*	ctDNA is a biomarker of response to kinase inhibitor therapy and it can be used to monitor resistance to treatment.
2015, Maria Gonzalez-Cao [95]	Cohort	Melanoma	22	TaqMan assay PCR	*BRAF*	Detection and accurate quantification of low- *BRAF* V600E in ctDNA can predict treatment outcome.
2016, Ademi Santiago-Walker [96]	Cohort	Melanoma	732	PCR-based BEAMing	*BRAF*	*BRAF* mutation using ctDNA analysis can be detected in >75% of patients and is a prognostic marker.
2016, Max Schreuer [97]	Cohort	Melanoma	36	qPCR	*BRAF*	Quantitative analysis of *BRAF* mutation in ctDNA is a monitoring tool during treatment with BRAF/MEK inhibitors.
2017, Stephen Q. Wong [98]	Cohort	Melanoma	52	Amplicon sequencing, ddPCR	*NRAS*, *BRAF*	ctDNA is a powerful complementary modality to functional imaging for real-time monitoring of tumor burden and genomic changes throughout therapy.
2018, R. Jeffrey Lee [99]	Cohort	Melanoma	161	QX200 ddPCR	*NRAS*, *BRAF*	ctDNA predicts relapse and survival in high-risk resected stage II/III melanoma and can help select patients for adjuvant therapy.
**Sarcoma**						
2016, Manuela Krumbholz [101]	Cohort	Ewing	20	AccuPrime Taq DNA PCR	*EWSR1-FLI1* fusion	Detection of EWSR1 fusion sequence in plasma is a promising noninvasive biomarker for improved therapeutic monitoring.
2016, Masanori Hayashi [102]	Cohort	Ewing	3	ddPCR	EWS-ETS	Tumor specific EWS-ETS translocation breakpoints in plasma DNA is a highly personalized biomarker for relapsed disease.
2018, David S. Shulman [103]	Cohort	Ewing, osteosarcoma	166	Illumina HiSeq 2500	EWSR1, FUS, CIC, CCNB3, TP53, STAG2	Detectable ctDNA in patients with localized disease is associated with inferior event-free survival and OS at 3 years compared to patients with undetectable ctDNA levels.
2013, Jacqueline Maier [104]	Prospective cohort	Gastrointestinal stromal tumor	38	RT-PCR	CKIT, PDGFRA	ctDNA harboring *CKIT* or *PDGFRA* was correlated with disease course.
2014, Changhoon Yoo [105]	Cohort	Gastrointestinal stromal tumor	30	PCR-based BEAMing	*CKIT, PDGFRA, BRAF*	Genotyping of the KIT gene in exon 17 of serum ctDNA identified mutations associated with resistance to dovitinib.
2016, Noriko Wada [106]	Cohort	Gastrointestinal stromal tumor	4	Sanger sequencing, PCR	*C-KIT*	Detection of secondary C-KIT mutations in ctDNA is useful to select targeted agents and to predict antitumor effects.
2018, Nicholas C. Eastley [107]	Cohort	Soft tissue sarcoma	11	Ion AmpliSeq	57 cancer-related genes	ctDNA analysis detected TP53/PIK3CA mutations concordant with the primary tumor in 2 of 4 cases.
**Brain tumors**						
2013, Mohamad A. Salkeni [108]	Prospective	Glioblastoma	13	Illumina HiSeq	EGFR (vIII deletion)	ctDNA analysis identified the EGFRvIII deletion in 3 of 13 patients, which was correlated with tumor tissue analysis and may help select patients for targeted therapy. ctDNA levels were correlated with the extent of tumor.
2016, Shigeki Yagyu [109]	Retrospective	Neuroblastoma	151	Real-time quantitative PCR	MYCN	Serum MYCN amplification (sensitivity 86%, specificity 95% compared with tissue analysis) was associated with OS. It may help select treatment prior to tumor biopsy, particularly for patients < 18 months (risk assessment and treatment depend on MYCN amplification status).
2012, Blandine Boisselier [110]	Prospective	Glioma	80 patients, 31 controls	Digital PCR, Agilent technologies	*IDH1*	The *IDH1* R132H mutation was identified in plasma (specificity, 100%; sensitivity related to the tumor volume and contrast enhancement). It may help in the diagnosis of patients not amenable to biopsy.
**Lymphoma**						
2016, Vincent Camus [111]	Cohort	Hodgkin	94	TaqMan assay PCR	XPO1	The XPO1 E571K mutation in ctDNA can be used as a novel biomarker in diagnosis and detection of minimal residual disease.
2016, Sarit E. Assouline [112]	Phase 2 trial	Diffuse large B-cell	40	ddPCR	CREBBP, EP300, MLL2, FAS, STAT6, TP53	Increase in ctDNA levels at 15 days after treatment initiation was associated with resistance to treatment.
2016, Florian Scherer [113]	Case control	Diffuse large B-cell	92 patients, 24 controls	CAPP-Seq	BCL2, BCL6, MYC, IGH	ctDNA levels at diagnosis were strongly correlated with clinical indices and were independently predictive of patient outcomes.

2015, David M. Kurtz [114]	Prospective cohort	Diffuse large B-cell	75	RT-PCR	Immunoglobulin high-throughput sequencing (Ig-HTS)	ctDNA immunoglobulin high-throughput sequencing preceded radiologic evidence of recurrent disease indicating that it may be a surrogate marker for monitoring disease after complete remission.
2015, Mark Roschewski [115]	Retrospective analysis	Diffuse large B-cell	126	LymphoSIGHT™	VDJ	After first-line treatment, disease progression was evident on imaging studies a median of 3.5 months after detection on ctDNA analysis of the clonal Ig gene sequence.
**Across Tumor Types**						
2012, Geraldine Perkins [117]	Cohort from multiple phase 1 trials	Colorectal, breast, melanoma, prostate, ovarian, and other	105	Sequenom MassArray, OncoCarta PCR	KRAS, BRAF, PIK3CA	ctDNA analysis has potential clinical multi-purpose utility in patients with advanced cancer.
2015, Filip Janku [118]	Cohort from multiple phase 1 trials	Colorectal, melanoma, non-small cell lung, and other	157	PCR-based BEAMing	BRAF, EGFR, KRAS, PIK3CA	Patients with > 1% of mutant ctDNA had shorter median OS compared to patients with ≤ 1%.
2014, Chetan Bettegowda [119]	Cohort	Pancreatic, ovarian, colorectal, bladder, gastroesophageal, breast, melanoma, hepatocellular, head and neck, and other	640	BEAMing, PCR-Ligation, Safe-SeqS	187 cancer-related genes	ctDNA is a broadly applicable, sensitive, and specific biomarker that can be used for clinical and research purposes in patients with various tumor types.
2013, Muhammed Murtaza [120]	Cohort	Breast, ovarian and lung	6	HiSeq 2500 Illumina	*PIK3CA, RB1, GAS6, EGFR*	ctDNA analyses can complement invasive tumor biopsies to identify mutations associated with acquired drug resistance in advanced cancer.
2015, Jean Sebastien Frenel [121]	Cohort	Colorectal, ovarian, breast, bladder, glioblastoma, lung adenocarcinoma, endometrial	39	Ion AmpliSeq, ddPCR	Cancer-related genes	Targeted sequencing of ctDNA has potential clinical utility to monitor the effect of targeted therapies.
2016, Maria Schwaederle [122]	Cohort	Lung, breast, glioblastoma, genitourinary, gastrointestinal, of unknown primary, and other	171	Illumina Hi-Seq 2500	54 cancer-related genes	A large proportion of patients had detectable ctDNA aberration (s), among which the majority are targetable by an approved drug.
2016, Maria Schwaederle [123]	Cohort	Brain, lung, breast	168	Illumina Hi-Seq 2500	54 cancer-related genes	ctDNA tests provide information complementary to the tissue biopsies and may be useful in determining prognosis and treatment.
2017, Yulian Khagi [124]	Cohort	Skin, lung, breast, glioblastoma, genitourinary, gastrointestinal, and other	69	Illumina Hi-Seq 2500	54–70 cancer-related genes	Hyper-mutated ctDNA is correlated with response to checkpoint inhibitor-based therapy and investigation of hypermutated ctDNA as a predictive biomarker is warranted.

Abbreviations: AR: Androgen Receptor; ddPCR: droplet digital PCR; FISH: fluorescence *in situ* hybridization; IHC: immunohistochemistry; mCRPC = metastatic castration-resistant prostate cancer; Met: methylation; MRD: minimal residual disease; NCT: neoadjuvant chemotherapy; NGS: next-generation sequencing; NSCLC: non-small-cell lung carcinoma; OS = overall survival; PCR: Polymerase chain reaction; PFS: Progression free survival; qPCR: quantitative polymerase chain reaction; rtPCR: real-time Polymerase chain reaction; SCC: Squamous cell carcinoma; SNVs: single nucleotide variants.

## CONCLUSIONS

ctDNA analysis is a non-invasive, cost-effective test with a potentially significant role in the early detection and diagnosis of tumors. Evolving data from clinical trials indicate the association of ctDNA with tumor burden and the usefulness of ctDNA analysis in assessment of MRD, in understanding mechanisms of resistance to treatment, and in dynamically guiding therapy. The discordance between ctDNA analysis and tumor tissue genomic analysis is attributed, at least in part, to biologic and technical differences in detection of alterations between DNA shed by the tumor in the circulatory system and DNA in tumor tissue. ctDNA is thought to reflect tumor from all sites of disease and is secreted by tumor cells, phagocyte-engulfed tumor cells, and necrotic or apoptotic tumor cells. Selected prospective trials with targeted agents incorporate ctDNA analysis to select targeted therapy. Longitudinal ctDNA analysis starting at the time of diagnosis will enrich our knowledge of the evolution of patients’ tumor biology, will accelerate drug development, and will contribute to the implementation of precision medicine to improve clinical outcomes.
